# The effectiveness of a mobile intervention to reduce young adults’ alcohol consumption to not exceed low-risk drinking guidelines

**DOI:** 10.3389/fdgth.2022.1016714

**Published:** 2022-12-06

**Authors:** Mieke H.J. Schulte, Nikolaos Boumparis, Annet Kleiboer, Tim R. Wind, Miranda Olff, Anja C. Huizink, Heleen Riper

**Affiliations:** ^1^ Department of Clinical, Neuro and Developmental Psychology, Amsterdam Public Health Research Institute, Vrije Universiteit, Amsterdam, Netherlands; ^2^ Foundation Centrum ‘45, partner in Arq Psychotrauma Expert Group, Diemen, Netherlands; ^3^ Arq Psychotrauma Expert Group, Diemen, Netherlands; ^4^ Department of Psychiatry, Amsterdam University Medical Centers Location AMC, Amsterdam Public Health, Amsterdam, Netherlands; ^5^ Department of Psychiatry, Amsterdam UMC-VUmc, Vrije Universiteit, Amsterdam, Netherlands

**Keywords:** young adults (18–29 years), alcohol, e-health, smartphone, RCT - randomized controlled trial, low-risk drinking guidelines

## Abstract

**Background:**

Young adults’ drinking habits often exceed low-risk drinking guidelines. As young adults show increased access, use, and interest in personalized content related to physical and mental well-being, mobile applications might be a suitable tool to reach this target group. This study investigates the effectiveness of “Boozebuster”, a self-guided mobile application incorporating various therapeutic principles to reduce young adults’ alcohol consumption to not exceeding low-risk drinking guideline levels, compared to an educational website condition.

**Method:**

Young adults aged 18–30 wanting to reduce their alcohol consumption entered a two-arm, parallel-group RCT. There were no minimum drinking severity inclusion criteria. Primary outcomes included alcohol consumption quantity and frequency. Secondary outcomes included binge drinking frequency and alcohol-related problem severity. Baseline, 6-week postbaseline, and 3-month post-baseline assessments were analyzed using linear mixed model analyses. Sex, treatment adherence, experienced engagement and motivation to change alcohol use behavior were investigated as moderators. Sub-group analyses contained problem drinkers and binge drinkers.

**Results:**

503 participants were randomized to the intervention or control condition. Results showed no intervention effects on primary or secondary outcomes compared to the control group. Both groups showed within-group reductions on all outcomes. Sub-group analyses in problem drinkers or binge drinkers showed similar results. Motivation to change drinking behavior and experienced engagement with the application significantly moderated the intervention effect regarding the quantity or frequency of alcohol consumption, respectively. Exploratory analyses showed that participants who indicated they wanted to change their drinking patterns during the initial PNF/MI module showed a significantly greater reduction in drinking quantity compared to those who indicated not wanting to change their drinking patterns.

**Conclusion:**

The intervention group did not show a greater reduction in alcohol-related outcomes compared to the control group, but both groups showed a similar decrease. Potential explanations include similar effectiveness of both condition due to using a minimal active control in participants predominantly in the action stage of motivation to change. Future research should further explore the effectiveness of using mobile application to reduce young adults’ drinking behavior to not exceed low-risk drinking guideline levels and identify factors that motivate participants to engage with such an intervention.

## Introduction

Alcohol consumption in young adults is widespread and a major health issue associated with physical, psychological, and social harm (1). Low-risk drinking guidelines have been developed in various countries and are aimed at moderating alcohol consumption and limiting alcohol-related harm (2, 3). For example, recent Dutch drinking guidelines (4) recommend not drinking at all or at least not more than one alcoholic drink per day for young and older adults. However, a substantial proportion of young and older adults commonly exceed low-risk drinking guidelines (5). About 8.9% of young adults are considered problem drinkers (6), defined as individuals who drink more than 14 or 21 standard glasses containing 10 g of ethanol per week for females or males, respectively. In addition, binge drinking (consuming at least 5 drinks during one occasion) occurs in about 11.4%–19.4% of young adults (7). Brief interventions such as personalized normative feedback (PNF) (8) have been found to be effective in reducing problematic alcohol use in young and older adults (9). To effectively reduce risky drinking behavior in young adults, ways to deliver these interventions to this target population need to be developed.

Exceeding low-risk drinking levels causes serious health problems (10) and a heavy social burden (11). Besides short-term and long-term morbidity and mortality (12), the consequences include accidents, sexual and physical assault, vandalism, and poor academic or work performance (1). Therefore, early identification and brief interventions have been increasingly reported to be cost-effective strategies to reduce problem drinking (9, 11, 13, 14). Evidence is most robust for brief interventions delivered in primary and secondary care (15–17). However, effectiveness has also been shown in the general population (18, 19) and university students (20, 21). Unfortunately, the implementation of brief interventions is hampered due to the limited availability of trained professionals, the difficulty of reaching problem drinkers, and the high costs of implementation and delivery (22, 23). Consequently, as many as 80% of problem drinkers are not receiving support (24, 25).

Digital interventions focusing on health and mental well-being are commonly used by young adults and have seen an increased uptake during the COVID-19 pandemic (26–28). Various digital interventions aimed at reducing drinking levels in young adults, mostly in university settings, have been evaluated. The majority of these interventions consist of internet-based single-session interventions based on PNF (8), general alcohol education programs (29), or brief interventions (30) that produce small but significant effect sizes ranging from g = 0.18 to g = 0.29 (31–36). Most of the above-described digital interventions are delivered *via* a computer or web browser. It has been claimed that mobile applications might be more suitable for young adults given their flexibility, interactivity, and spontaneous nature (37). Although many mobile applications claiming to effectively reduce alcohol consumption are currently available in commercial app stores, the number of evidence-based mobile applications is limited and show mixed findings (38). More specifically, Colbert et al. (38) reviewed 12 studies on smartphone applications designed for adults from the general population or with an alcohol use disorder. Of these studies, 5 were RCTs of which 2 studies reported significant reductions in alcohol consumption outcomes compared with active or inactive control groups and 3 studies reported no differences in alcohol consumption outcomes compared with control groups.

Despite the potential benefits of using a mobile application, young adults show limited interest in receiving solely alcohol-related information as an intervention to reduce alcohol consumption (39). This could negatively affect their interest in such an intervention and consequently lead to reduced recruitment and engagement rates. To the best of our knowledge, this is the first smartphone application that has been developed in the Netherlands to reduce alcohol consumption to not exceed the low-risk guidelines in young adults. Given that young adults show increased access, use, and interest in healthy lifestyle related content delivered through mobile applications (40, 41), we have chosen a health promotion approach in developing the mobile application in order to make the application more appealing.

This study aimed to investigate the effectiveness of a mobile application (“Boozebuster”) in reducing young adults’ alcohol consumption to not exceed low-risk drinking guidelines. It is hypothesized that the intervention group will show a greater reduction in the alcohol-related outcome measures than the minimal active control group. In addition, potential factors that could moderate the effectiveness of digital interventions have been investigated such as motivation (42), adherence (43), and engagement (44) of the users. Moreover, research on treatment for problem drinking has predominantly focused on men, resulting in suboptimal interventions for women. Therefore, it is important to investigate if there are sex-dependent differences in the effectiveness of digital interventions in reducing alcohol consumption.

## Materials and methods

### Study design

We conducted a two-arm, parallel-group, randomized controlled trial (RCT) comparing a 6-week self-guided mobile application (“Boozebuster”) and compared it with an educational website condition containing information on the effects and health consequences of alcohol use. Due to the nature of the study design, blinding of group allocation was not possible. Detailed information about the study design is provided in the study protocol (45).

### Randomization

Participants were randomized to receive either the intervention condition or the minimal active control condition. The allocation sequence was automatically generated *via* the computerized Castor EDC system using a 1:1 allocation ratio with random block sizes of 4 and 6. Stratification was based on sex and adherence to the Dutch drinking guidelines (drinking more or less than seven drinks per week and/or having at least one binge-drinking session in the past 30 days or not).

### Sample size and power

Based on the individual patient data meta-analysis of Riper et al. (46) assessing brief interventions to reduce alcohol consumption in young adults compared to minimal-intervention controls, an effect size of g = 0.25 was deemed appropriate. With an *α *= .05 and 1−*β *= .80, these estimations resulted in a sample size of 253 per study arm (i.e., *N* = 506).

### Participants

Recruitment took place between January 2021 and May 2021 *via* Facebook advertisements or an online recruitment website (i.e., Link2Trials.com). Inclusion criteria were: aged between 18 and 30 years, willingness to reduce drinking behavior as part of a healthy lifestyle, proficiency in reading and writing in Dutch, access to an Android or iOS device with a connection to the internet, and possession of an email address. A minimum drinking severity inclusion criterion was not applied as we chose a health promotion approach in which everyone interested in improving their health, including reducing their alcohol consumption, could participate. No exclusion criteria were applied.

### Study procedure

The Scientific and Ethical Review Committee of the Faculty of Behavioral and Movement Sciences of the Vrije Universiteit Amsterdam approved the study protocol (VCWE-2018-045). The study was registered at the Netherlands Trial Register (NL8828). Individuals, who visited our study information website and were interested in participating, were screened on inclusion criteria on the website. Online informed consent was obtained from all participants after a complete description of the study. Those who met the criteria were entered into the computerized Castor Electronic Data Capturing (EDC) system (47) and received a link to fill out the baseline (T0) questionnaires. Participants were randomized to receive the intervention or the educational website. When allocated to the intervention condition, a link was sent to download the mobile application with a short description on how to use the app during the next 6 weeks. When allocated to the educational website condition, participants received a link directing them to the educational website. All participants were offered an incentive of €10 to complete the post-intervention assessment (T1) assessment and an additional €10 to complete the 3-month follow-up (T2) assessment.

### Boozebuster intervention

Boozebuster contains seven modules focusing on alcohol use and multiple behavioral change techniques that could help to alter drinking patterns. The core components of the intervention focus on reducing drinking habits and incorporate therapeutic principles including personalized normative feedback (PNF) (8), motivational interviewing (MI) (48) and protective behavioral strategies (PBS) (49). Additional components that could facilitate low-risk drinking habits were focused on for example improving sleep and relieving stress.

The developmental process included several steps involving the potential end-users, which included interviews, brainstorm sessions and focus groups. First, students were interviewed on the need and desirability of a smartphone application to reduced alcohol consumption in young adults. Next, we brainstormed with several e-health experts on how to incorporate interactive components to the app and how to make it more attractive. Based on the acquired input, a conceptual framework for the application and feasible recruitment strategies were developed by the research team. The prototype was further evaluated in a focus group of potential end-users (i.e., representatives from the age-group ranging from 18 to 30 years old), after which the application was further adjusted to meet their preferences as expressed in the focus group. As a last step, a technical pilot was conducted (*n* = 15) to spot possible bugs and errors.

All participants started with the mandatory module containing PNF (36) and MI (48). In this module, drinking patterns were assessed and compared with peer norms and official Dutch drinking guidelines to create awareness about participants’ alcohol consumption compared to their peers and the guidelines. Participants were asked if they wanted to alter their drinking habits during the PNF module. If they responded positively, MI components followed with the intent to intrinsically motivate participants to change their drinking habits. On the other hand, if participants responded negatively, the module ended while thanking the participants for their time.

Subsequently, participants could choose which module they would like to follow and were able to revisit each module as desired. In the module focusing on goals, participants could select a goal they wished to achieve related to alcohol consumption, such as saving money, living healthier, or coping with peer pressure. Each goal was accompanied by a set of recommendations to achieve that goal. The mindfulness module presented participants with four guided meditations to relieve stress: a body scan, sleep meditation, urge surfing, and muscle relaxation. The sleeping module gave participants advice on improving their sleep quality. In the diary module, participants received daily notifications randomly between 9 AM and 9 PM to indicate the number of drinks consumed yesterday and rate their mood and sleep on a scale from 0 to 10. Responses were displayed as visual feedback enabling participants to monitor their progress. In addition, participants could report what they were grateful for in the gratitude diary. The final module contained an emergency button, providing recommendations based on protective behavioral strategies for acute cravings (49). In addition, these strategies could be modified by the participants by adding or removing strategies suitable for their individual needs.

### Educational website condition

Participants allocated to the educational website condition received a link redirecting them to an educational website containing information on the effects and health consequences of alcohol use, without the additional components that were added in the app. The included information is commonly used for psychoeducation purposes and was slightly more extensive compared to the consequences of excessive alcohol consumption that were provided in the app. Participants in the control condition could revisit the website whenever they wanted.

### Assessments

Participants of both groups were invited to fill out questionnaires at baseline (T0), 6-week post-intervention (T1), and 3-month follow-up (T2). Sociodemographic variables were assessed at baseline, including age, sex, education level, employment, and marital status. The key outcomes of this study are based on the recommendation of the Outcome Reporting in Brief Intervention Trials on Alcohol (ORBITAL) framework (50). An overview of the assessments at each time point is presented in Table 1.

**Table 1:** Overview of measurements, timepoints and instruments.

Outcome measures	Baseline	6-week post-test	3-month follow-up
Sociodemographics	x		
Alcohol consumption, frequency and quantity (TLFB)	x	x	x
Binge drinking frequency	x	x	x
Alcohol-related problem drinking (RAPI)	x	x	x
Participants’ motivation (RCQ)	x	x	
Treatment adherence		x	
Engagement (TWEETS)	x	x	

RAPI, Rutgers Alcohol Problem Index; RCQ, Readiness to Change Questionnaire; TLFB, TimeLine Follow Back; TWEETS, TWente Engagement with Ehealth Technologies Scale.

#### Primary outcomes

As the intervention could affect the quantity as well as the frequency of alcohol consumption, the primary outcomes of this study are the frequency and quantity of alcohol consumption, as measured in standard units containing 10 g of ethanol. The frequency and quantity of alcohol consumption were measured using the TimeLine Follow-Back questionnaire (TLFB) (51) by asking to report the number of standard units consumed per day for the past 30 days.

#### Secondary outcomes

Binge drinking frequency was assessed by asking participants the number of days they consumed 4 (for females) or 5 (for males) standard units on one occasion during the past 30 days. Alcohol-related problem severity was assessed using the Rutgers Alcohol Problem Index (RAPI) (52).

#### Moderators

Several potential moderators were assessed that could affect the effect size regarding the primary outcomes, including sex, motivation to change alcohol use behavior, engagement with the intervention (44), and treatment adherence (43). Baseline motivation to change alcohol use behavior was assessed using the Readiness to Change Questionnaire (RCQ) (53). Since only the intervention group received the mobile application, engagement with the intervention and intervention adherence could only be assessed in the intervention group. Experienced engagement with the intervention was assessed using the Twente Engagement with EHealth Technologies Scale (TWEETS) (54). Intervention adherence was calculated descriptively based on the percentage of participants who completed the main PNF/MI module.

### Statistical analyses

Data analyses were performed using IBM SPSS version 27. Baseline between-group differences of continuous data were analyzed using independent-samples t-tests. Categorical data were analyzed using chi-square tests.

To analyze the effects on the primary and secondary outcomes, time (T0 vs. T1 vs. T2) by group (Boozebuster vs. educational website condition) Linear Mixed Model (LMM) analyses were conducted. LMM analysis takes the dependence between observations due to clustering of data by participants into account and allows for the number of observations between participants to be different (55). For all LMM analyses, a random intercept was used to correct the dependency of repeated measures. In addition, a random slope was included when the likelihood ratio test indicated that this was an improvement on the model. In all LMM analyses, the residuals were checked for normal distribution. Data were analyzed using the intention to treat (ITT) principle, as well as by using sensitivity analyses by the per-protocol (i.e., completed the first PNF/MI module) and the study completers analysis principle. Exploratory analyses were performed to investigate if the intervention might have been more effective for participants presenting with a particular drinking pattern, including problem drinkers (drinking more than 14/21 units per week for women/men), and binge drinkers (drinking more than 4/5 units per occasion for women/men in the last 30 days).

To investigate their moderating effect on the primary outcome measures, sex, motivation to change alcohol use behavior, engagement with the intervention, and treatment adherence were added as a factor in the LMM analyses. As engagement with the intervention and treatment adherence could only be assessed in the intervention group, moderation analyses regarding these measures were only performed in the intervention group.

## Results

### Participants

**Figure 1:**
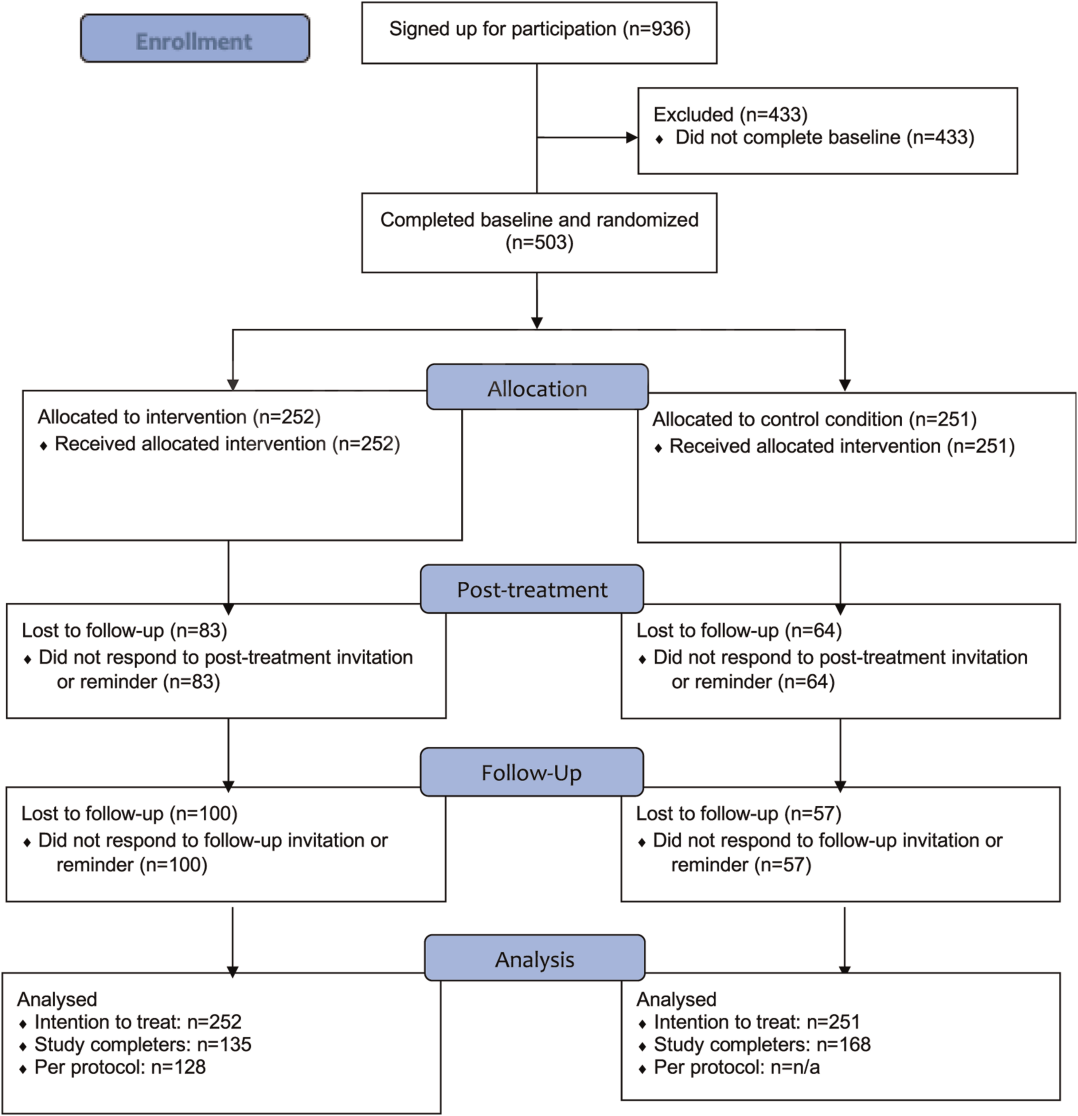
CONSORT flow diagram. Participants who did not complete post-treatment assessment were still allowed to complete the follow-up assessment.

A total of 936 individuals met the inclusion criteria and were entered into the electronic data capturing system. Of these 936, 503 participants provided informed consent, completed the baseline questionnaires, and were randomized to the intervention condition (*n* = 252) or control condition (*n* = 251). Due to clerical errors, three participants were entered in the system twice, resulting in the randomization of 503 participants instead of 506. Of the 503 participants, 356 (70.8%) completed the T1 assessment, whereas 346 (68.8%) completed the T2 assessment. It must be noted that not all participants who completed the T2 assessment also completed the T1 assessment, and vice versa. More specifically, 104 (20.7%) participants did not complete any post-baseline assessments, 96 participants (19.1%) completed one assessment (T1 or T2), and 303 (60.2%) completed both assessments. Of the participants who completed only one assessment, 53 (55.2%) completed only the T1 assessment, whereas 43 participants (44.8%) completed only the T2 assessment. See Figure 1 for the CONSORT FLOW chart.

Baseline characteristics are summarized in Table 2. At baseline, there were no between-group differences in demographic characteristics, including age, sex, education, and occupation. However, there was a between-group difference in marital status, with more participants being in a relationship than being single in the control condition (58.2% vs. 41.8%) compared to the intervention condition (51.2% vs. 48.8%). In addition, there were no between-group differences in alcohol use characteristics.

**Table 2:** Baseline characteristics of participants who were randomized after completing the baseline assessment.

	All (*n* = 503)	Boozebuster (*n* = 252)	Control group (*n* = 251)	t (df)^a^/*χ*^2^ (df)^b^	p-value
Randomization, *n* (%)		–	–	–	–
Boozebuster	252 (50.1)				
Control	251 (49.9)				
Age	22.98 (3.39)	22.89 (3.27)	23.06 (3.50)	0.553 (501)^a^	.68
Sex, *n* (%)				.000 (1)^b^	.99
Female	441 (87.7)	221 (87.7)	220 (87.6)		
Male	62 (12.3)	31 (12.3)	31 (12.4)		
Education, *n* (%)				2.728 (3)^b^	.44
Primary school	3 (0.6)	2 (0.8)	1 (0.4)		
Secondary school	99 (19.7)	44 (17.5)	55 (21.9)		
Vocational training	28 (5.6)	12 (4.8)	16 (6.4)		
Higher education	373 (74.2)	194 (77.0)	179 (71.3)		
Occupation, *n* (%)				.600 (2)^b^	.74
Student	313 (62.2)	160 (63.5)	153 (61.0)		
Working	167 (33.2)	82 (32.5)	85 (33.9)		
Not working	23 (4.6)	10 (4.0)	13 (5.2)		
Marital status, *n* (%)				4.426 (1)^b^	**.04**
Single	234 (46.5)	129 (51.2)	105 (41.8)		
In a relationship	269 (53.5)	123 (48.8)	146 (58.2)		
Alcohol use					
Drinker, *n* (%)	494 (98,2)	248 (98.41)	246 (98.01)	.117 (1)^b^	.73
Drinks/month	50.02 (44.54)	49.11 (42.22)	50.93 (46.82)	0.458 (501)^a^	.65
Days/month	10.06 (6.01)	9.95 (5.81)	10.17 (6.21)	0.408 (501)^a^	.68
Risky drinker, *n* (%)	410 (81.5)	205 (81.3)	205 (81.7)	0.009 (1)^b^	.93
Binge drinker, *n* (%)	400 (79.5)	199 (78.97)	201 (79.76)	.095 (1)^b^	.76
Binge frequency	3.37 (3.58)	3.32 (3.41)	3.42 (3.76)	0.315 (501)^a^	.75
RAPI	28.89 (8.12)	28.67 (7.83)	29.11 (8.42)	0.603 (501)^a^	.55
RCQ (phase)				.011 (2)^b^	.99
Precontemplation, *n* (%)	69 (13.7)	35 (14.0)	34 (13.7)		
Contemplation, *n* (%)	182 (36.2)	91 (36.4)	91 (36.7)		
Action, *n* (%)	247 (49.1)	124 (49.6)	123 (49.6)		
TWEETS^a^					
Total	24.49 (4.83)	24.39 (5.24)	24.59 (4.38)	0.457 (501)^a^	.65
Behavioral engagement	8.01 (1.68)	7.95 (1.78)	8.08 (1.57)	0.876 (501)^a^	.38
Cognitive engagement	8.39 (2.01)	8.32 (2.20)	8.47 (1.80­)	0.807 (501)^a^	.42
Affective engagement	8.08 (2.03)	8.12 (2.12)	8.04 (1.94)	−0.437 (501)^a^	.66

The bold values indicate significance. CES-D, Center for Epidemiologic Studies Depression Scale; RAPI, Rutgers Alcohol Problem Index; RCQ, Readiness to Change Questionnaire; TWEETS, TWente Engagement with Ehealth Technologies Scale.

^a^ TWEETS was assessed at post-intervention assessment (T1).

### Primary outcomes

There was no main effect of group nor an interaction effect of group X time on either the quantity or the frequency of alcohol consumption. However, there was a significant effect of time on both the quantity and frequency of alcohol consumption in both groups. Regarding alcohol consumption quantity, both groups showed a decrease of around 17 units/month at T1 that slightly increased towards T2. Similar for alcohol consumption frequency, both groups showed a decrease of around 2 days per month at T1, but only the control group showed a slight increase towards T2 (see Table 3). Sensitivity analyses (i.e., per protocol and study completers analyses) resulted in similar results regarding the primary outcome variables as the ITT analyses (see Table 4).

**Table 3:** Effect of Boozebuster app on primary and secondary outcome measures.

	Boozebuster			Control			Effects								
	T0	T1	T2	T0	T1	T2	Time			Group			Group X time		
	*n* = 252	*n* = 184	*n* = 153	*n* = 251	*n* = 202	*n* = 196	F (df)	*p*	d	F (df)	*p*	d	F (df)	*p*	d
Alcohol use															
Drinks/month	49.11 (42.22)	31.80 (33.83)	32.96 (41.02)	50.93 (46.82)	32.62 (36.00)	35.85 (38.37)	69.813 (395.75)	**<0.001**	0.75	0.042 (480.20)	.84	0.02	0.467 (395.75)	.5	0.06
Days/month	9.95 (5.81)	7.91 (6.11)	7.91 (6.51)	10.17 (6.21)	7.61 (5.60)	7.99 (6.24)	57.644 (386.19)	**<0.001**	0.68	0.058 (490.66)	.81	0.02	0.046 (386.19	.83	0.02
Binge freq.	3.32 (3.41)	2.18 (2.97)	2.34 (3.13)	3.42 (3.76)	2.32 (3.54)	2.55 (3.60)	25.950 (768.78)	**<0.001**	0.46	0.000 (1165.05)	.98	0.001	1.010 (768.78)	.32	0.09
RAPI	28.67 (7.83)	25.45 (7.26)	24.09 (6.90)	29.11 (8.42)	25.87 (7.33)	24.58 (6.82)	149.861 (398.86)	**<0.001**	1.09	0.170 (485.39)	.68	0.04	0.058 (398.86)	.81	0.02

The bold values indicate significance. RAPI, Rutgers Alcohol Problem Index.

**Table 4:** Sub-group analyses on the effect of Boozebuster app on primary outcome measures.

	Boozebuster			Control			Effects								
	T0	T1	T2	T0	T1	T2	Time			Group			Group X time		
Drinking Subgroups							F (df)	*p*	d	F (df)	*p*	d	F (df)	*p*	d
Study completers (135/168)															
Drinks/month	44.95 (37.93)	27.79 (28.59)	33.04 (41.72)	44.32 (38.87)	31.26 (32.30)	32.62 (32.41)	39.187 (301.000)	**<0.001**	0.73	0.012 (301.000)	.91	0.01	0.003 (301.000)	.96	0.01
Days/month	9.56 (5.79)	7.47 (6.09)	7.96 (6.45)	9.31 (5.47)	7.38 (5.26)	7.54 (31.64)	39.826 (301.000)	**<0.001**	0.73	0.013 (301.000)	.91	0.01	0.096 (301.000)	.76	0.04
Intervention completers (128/251)															
Drinks/month	57.86 (43.66)	36.24 (38.16)	36.61 (44.78)	50.93 (46.82)	32.62 (36.00)	35.85 (38.27)	69.813 (395.750)	**<0.001**	0.91	0.042 (480.200)	.84	0.02	0.467 (395.750)	.50	0.07
Days/month	10.95 (5.80)	8.38 (6.01)	8.12 (6.21)	10.17 (6.21)	7.61 (5.60)	7.99 (6.24)	57.644 (386.189)	**<0.001**	0.83	0.058 (490.655)	.81	0.03	0.046 (386.189)	.83	0.02
Problem drinkers (120/164)															
Drinks/month	55.61 (38.19)	32.90 (28.75)	39.11 (43.52)	56.40 (43.93)	38.76 (37.92)	41.37 (39.92)	79.415 (624.324)	**<0.001**	1.07	0.225 (931.177)	.64	0.06	0.222 (624.324)	.64	0.06
Days/month	11.08 (5.54)	8.66 (5.87)	9.18 (6.34)	11.18 (5.42)	8.68 (5.67)	8.98 (6.30)	58.480 (306.846)	**<0.001**	0.92	0.953 (396.258)	.33	0.12	0.220 (306.846)	.64	0.06
Binge drinkers (119/141)															
Drinks/month	53.98 (38.98)	33.21 (28.77)	39.18 (43.45)	55.00 (45.20)	37.90 (38.49)	41.58 (40.22)	164.282 (221.902)	**<0.001**	1.6	0.070 (596.268)	.79	0.03	0.037 (221.902)	.85	0.02
Days/month	10.61 (5.74)	8.58 (5.99)	9.04 (6.24)	10.60 (5.89)	8.37 (5.84)	8.78 (6.49)	153.118 (208.612)	**<0.001**	1.55	0.199 (357.588)	.66	0.06	0.041 (208.612)	.84	0.03

The bold values indicate significance.

### Secondary outcomes

There was no main effect of group nor an interaction effect of group X time on binge drinking frequency and RAPI scores. However, there was a significant main effect of time on both binge drinking frequency and RAPI scores. Regarding binge drinking frequency, both groups showed a decrease of around 1 binge drinking day in the last 30 days at T1, that slightly increased towards T2. In addition, both groups showed a decrease of around 3 points on the RAPI at T1, that continued towards a decrease of around 4.50 points at T2 (see Table 3).

### Sub-group analyses

For both sub-group analyses (i.e., problem drinkers and binge drinkers), the results were identical to the ITT analyses with no between-group differences regarding the primary and secondary outcomes. More specifically, there was no main effect of group nor an interaction effect of group and time on the quantity or the frequency of alcohol consumption. However, both groups showed a decrease in the quantity and frequency of alcohol consumption over time (see Table 4). In the sub-group of problem drinkers, both groups showed a decrease in the quantity of alcohol consumption of around 20 units at T1, that slightly increased towards T2. In addition, both groups showed a decrease in alcohol consumption frequency of around 2.5 days per month at T1, that slightly increased towards T2. In the sub-group of binge drinkers, both groups showed a decrease in the quantity of alcohol consumption of around 20 units at T1, that slightly increased towards T2. In addition, both groups showed a decrease in alcohol consumption frequency of around 2 days per month at T1, that slightly increased towards T2. The results regarding all sub-group analyses are displayed in Table 4.

### Moderation analyses

There were no moderating effects of sex regarding alcohol consumption quantity [F(1, 760.46) = 1.935, *p* = 0.17, d = 0.12] or frequency [F(1, 777.03) = 0.784, *p* = 0.38, d = 0.08].

Intervention adherence did not moderate the intervention effect on alcohol consumption quantity [F(1, 388.34)= 2.339, *p* = 0.13, d = 0.14] or frequency [F(1, 384.79) = 1.263, *p* = 0.26, d = 0.13]. In addition, there was no moderating effect of the number of times the app was opened on alcohol consumption quantity [F(66, 200.99) = 1.172, *p* = 0.20, d = 1,24] or frequency [F(66, 204.14) = 1.003, *p* = 0.48, d = 1.14].

Baseline motivation to change alcohol use behavior did not moderate the intervention effect on the frequency of alcohol consumption [F(33, 317.79) = 1.402, *p* = 0.08, d = 0.76]. However, there was a three-way interaction between group, time, and baseline RCQ scores regarding the quantity of alcohol consumption [F(33, 698.01) = 1.539, *p* = 0.03, d = 0.54]. The intervention effect was compared between participants with low and high baseline RCQ scores based on a median split. It seemed that, in participants with higher RCQ scores, there was a greater reduction in the quantity of alcohol consumption in the intervention group compared to the control group. This effect was not present in participants with lower RCQ scores.

Engagement with the intervention did not moderate the intervention effect on alcohol consumption quantity [F(34, 336.52) = 1.015, *p* = 0.45, d = 0.64]. However, engagement did moderate the intervention effect on alcohol consumption frequency [F(34, 331.223) = 1.657, *p* = 0.01, d = 0.82]. The intervention effect was compared between participants with low and high T1 TWEETS scores based on a median split. Both groups seemed to show a similar reduction in alcohol consumption frequency at T1, but the group with lower engagement showed a greater increase at the 3-month follow-up compared to the group with higher engagement.

### Exploring the extent of app use

As the extent to which the app was used could substantially influence its effect on the outcome measures, additional exploratory analyses were performed to investigate if this was the case in this study. To this end, intervention effects were investigated in sub-groups that showed increasingly more extensive use of the app. The intervention effect was investigated for these sub-groups compared to the complete control group. Of the 252 participants, who were allocated to the intervention condition, 231 participants logged in to the app, and of those 161 opened the app at least once. Compared to the control group, there was no intervention effect in this group on the quantity of alcohol consumption [F(1, 347.36) = 0.527, *p* = 0.47, d = 0.07] or the frequency of alcohol consumption (F1, 349.51) = 0.009, *p* = 0.92, d = 0.01). Of these 161 participants, 128 participants completed the initial PNF/MI module. In this group there was no intervention effect on the quantity of alcohol consumption [F(1, 318.06) = 1.592, *p* = 0.21, d = 0.14] or the frequency of alcohol consumption [F(1, 318.64) = 0.821, *p* = 0.37, d = 0.10] compared to the control group. In the initial PNF/MI module, participants were asked if they wanted to alter their drinking patterns. Of the 128 participants who finished the module, 80 participants responded positively. Compared to the control group, there was an intervention effect on the quantity of alcohol consumption [F(1, 281.80) = 8.05, *p* < 0.01, d = 0.37], but not on the frequency of alcohol consumption [F(1, 280.141) = 3.43, *p* = 0.07, d = 0.24]. The participants who responded positively showed an average decrease in past 30-day consumption of 31.64 units from T0 to T2. In contrast, the control group showed an average decrease in past 30-day consumption of 15.08 units from T0 to T2. When comparing the participants who responded positively to the participants who responded negatively (*n* = 48), there also was an intervention effect on the quantity of alcohol consumption [F(1, 106.22) = 6.599, *p* = 0.01, d = 0.56], but not on the frequency of alcohol consumption [F(1, 218.24) = 3.726, *p* = 0.06, d = 0.42]. The participants who responded negatively showed an average decrease of 4.15 units from T0 to T2.

## Discussion

This study aimed to investigate the effectiveness of a mobile application (“Boozebuster”) in reducing young adults’ alcohol consumption to not exceeding low-risk drinking guideline levels. Compared to the minimal active control group, there was no significant effect of the Boozebuster application on the primary outcomes, i.e., the quantity and frequency of alcohol consumption, nor on the secondary outcomes, i.e., binge drinking frequency or alcohol-related problem severity. However, both groups showed a significant reduction over time regarding these alcohol-related outcome measures. Furthermore, Boozebuster was not more effective compared to the minimal active control group in sub-groups of problem drinkers or binge drinkers. In addition, there was no moderating effect of sex and adherence concerning the primary outcomes. However, baseline motivation to change alcohol use behavior significantly moderated the treatment effect regarding the quantity of alcohol consumption in that the intervention group showed a greater reduction compared to the control group when motivation was higher, but that this difference was absent when motivation was lower. Experienced engagement with the intervention significantly moderated the frequency of alcohol consumption in that, after showing a similar alcohol consumption frequency at the post-intervention assessment, the group with higher engagement showed a lower drinking frequency at the 3-month follow-up compared to the group with lower engagement. Exploratory analysis on the extent to which the app was used indicated that participants who responded positively towards altering their drinking patterns in the initial PNF/MI module of the app showed a greater decrease in alcohol consumption quantity, but not frequency, compared to control group or participants who responded negatively.

Although positive effects of mobile applications on alcohol consumption have been reported in previous RCTs (56, 57), the current findings showed no differential effects of the mobile application compared to the educational website condition on alcohol-related outcomes (58, 59). As sensitivity analyses provided identical results, the current findings appear robust. One explanation for our groups’ similar decrease in alcohol consumption might be the participants’ motivation to develop a healthier lifestyle, including reducing their alcohol consumption to not exceeding low-risk drinking guideline levels. As in both groups almost 50% of the participants were in the action stage of their readiness to change, participating in this study might have been enough to activate them to develop this healthier lifestyle regardless of which group they were allocated to. Moreover, although solely alcohol-related information is of limited interest to young adults (39), the educational website can be seen as a minimal intervention and therefore could have been equally effective in reducing drinking behavior compared to the intervention.

Several moderators were investigated in the current study, including sex, motivation to change alcohol use behavior, experienced engagement with the intervention, and adherence. Only motivation to change alcohol use behavior and experienced engagement appeared to moderate the treatment effect on the quantity or frequency of alcohol consumption, respectively, which is in line with a previous study that only found an effect of a mobile application on alcohol consumption in participants engaged with the mobile application (60). Interestingly, an intervention effect on alcohol consumption quantity, but not frequency, was found in participants who indicated they wanted to change their drinking patterns during the PNF/MI module compared to the control group or participants who indicated not to be willing to change. Similar findings of a decrease in alcohol quantity, while the frequency remains stable, have been reported for the general population and university students that received brief alcohol interventions (61, 62). One potential explanation could be that young adults experience it to be easier to decrease the amount of alcohol consumption rather than maintaining complete abstinence, for example due to social desirability.

Strengths of this study include the large sample size, the open recruitment strategy, and being the first smartphone application that has been developed in the Netherlands to reduce young adults’ alcohol consumption to not exceed low-risk drinking guideline levels. Limitations of this study include using incentives for completing the follow-up assessments. This could have resulted in the inclusion of participants unwilling to change their drinking behavior, as indicated in the initial PNF/MI module. Another limitation is applying self-report measures to assess changes over time. Nevertheless, self-report measures have been found to be reliable for actual drinking levels in our target population (63). In addition, as a result of the health promotion approach, not all participants were frequent drinkers. Consequently, not all participants were able to reduce their alcohol consumption as it was already minimal, resulting in a potential floor effect. Lastly, the education website could be seen as a minimal intervention, in which participants received information about the effects and consequences of alcohol consumption. This could have resulted in similar effects compared to the intervention.

As the health promotion approach appeared to be a successful recruitment strategy, future research could benefit from identifying the factors that motivate people to engage with an intervention such as the Boozebuster app. Moreover, including a qualitative assessment of participants’ experience regarding the use of the application could result in improved participants’ engagement to future interventions. Furthermore, it should be investigated if this mobile application could be useful as a preventative strategy in people who already show more severe alcohol consumption levels such as problem drinking. Furthermore, the timeline of our study happened to take place during the COVID-19 pandemic, which may have affected our target group’s drinking and social patterns, resulting in reductions in alcohol consumption that were not a result of participating in the study. In addition, our study sample consisted mainly of women (87.7%). Sex is an important health determinant and the over-representation of women in our sample might have led to sampling bias limiting the generalizability of our findings. Future studies should further investigate moderating variables, such as motivation to alter drinking habits, engagement with the intervention and their impact on the intervention effectiveness. A more comprehensive understanding of these variables could ultimately contribute to the development of more effective interventions.

This study did not find a differential effect of the Boozebuster application on alcohol-related outcomes compared to a minimal active educational website condition, but similar reductions in both groups. This could potentially be due to high motivation to develop a healthier lifestyle in both groups, leading to equal effectiveness of application and the educational website. Moreover, choosing a health promotion approach in which everyone interested in improving their health, including reducing their alcohol consumption could participate appeared to be a successful recruitment strategy. Making alcohol-related information available and appealing to young adults might be an important contribution to tackling alcohol-related harms and improving this target group’s well-being. Future efforts to examine interventions delivered *via* mobile applications will be critical to establishing the added value of such approaches in reducing young adults’ drinking behavior to not exceed low-risk drinking guideline levels.

## Data Availability

The meta-data is available and raw data will be shared upon reasonable request from the authors if in line with GDPR.
